# Long-term survival after stroke in Lithuania: Data from Kaunas population-based stroke registry

**DOI:** 10.1371/journal.pone.0219392

**Published:** 2019-07-10

**Authors:** Ricardas Radisauskas, Abdonas Tamosiunas, Daina Kranciukaite-Butylkiniene, Egle Milinaviciene, Vilija Malinauskiene, Gailute Bernotiene, Dalia Luksiene, Dalia Virviciute, Daiva Rastenyte

**Affiliations:** 1 Institute of Cardiology, Lithuanian University of Health Sciences, Kaunas, Lithuania; 2 Department of Environmental and Occupational Medicine, Lithuanian University of Health Sciences, Kaunas, Lithuania; 3 Department of Preventive Medicine, Lithuanian University of Health Sciences, Kaunas, Lithuania; 4 Department of Family Medicine, Lithuanian University of Health Sciences, Kaunas, Lithuania; 5 Department of Rehabilitation, Lithuanian University of Health Sciences, Kaunas, Lithuania; 6 Department of Neurology, Lithuanian University of Health Sciences, Kaunas, Lithuania; Chinese Academy of Medical Sciences and Peking Union Medical College, CHINA

## Abstract

**Background:**

There is a lack of reliable epidemiological data on long-term survival trends of first-ever stroke patients in Lithuanian population.

**Aims:**

To evaluate trends in long-term survival after stroke and to determine the influence of some sociodemographic and lifestyle factors, time and subtype of stroke, and stroke care on survival.

**Methods:**

All stroke events included in Kaunas stroke register database were ascertained and validated according to the standardized criteria outlined by the WHO MONICA Project. The study included all patients in Kaunas (Lithuania) city aged 25 to 64 years who experienced a stroke between 1986 and 2011. Death time was confirmed by the Office for National Death Statistics. Estimates of stroke long-term survival data and factors influencing survival changes were made by applying the Kaplan-Meier and Cox regression analysis.

**Results:**

During the study period, 4,129 persons aged 25–64 years suffered from a first-ever stroke: 2,215 (53.6%) of them were men and 1,914 (46.4%)—women. Ischemic stroke was significantly more frequent in males than in females (80.6% and 78.6%, respectively, p<0.05) and subarachnoid hemorrhage was more common in women than in men (9.0% and 7.0% respectively, p <0.05). Of all first-ever stroke patients, 3,272 (79.2%) survived 1 year and 2,905 (70.4%) survived 5 years after stroke onset. The 1- and 5-years survival rate after a first-ever stroke in women was significantly higher as compared with that in men (Log-rank test p = 0.0001). The older (55–64 year) persons had poorer 1-year and 5-years survival rate as compared with persons in the younger (25–54 years) age group (Log-rank test p = 0.0001). Among persons with a first-ever stroke who had their stroke in 2007–2011, 1- and 5-year survival rate was higher compared with that in persons who had had a stroke in 1986–1990 and in 1997–2001 (Log-rank test p = 0.0001). The persons with a first-ever ischemic stroke had a better chance to survive first 1- and 5-years after stroke compared with persons who had intracerebral or subarachnoid haemorrhage. Only female gender was associated with higher 1- and 5-year survival rate after first-ever stroke. The older age, previous myocardial infarction and diabetes mellitus were associated with lower 1- and 5-year survival rate after first-ever stroke.

**Conclusions:**

This population-based study of patients with first-ever stroke demonstrated that the long-term survival was better in women than men, and improved significantly in both men and women during the past decade. Long-term survival was better of those with first-ever ischemic stroke and of younger age– 25 to 54 years.

## Introduction

Worldwide stroke is the fifth leading cause of death in low-income countries and the second in high-income countries [1]. Stroke designates substantial burden, both economic and social, on individuals and families [2–5].

Mortality rates for stroke are reported to be declining in well developed countries, such as Western European countries, the USA, Canada, Australia, the United Kingdom and Spain, supposedly because risk reduction programmes are being implemented [6–12]. The decline in stroke mortality over the past decades, the major improvement in population health observed for both sexes and all age groups, have resulted from reduced stroke incidence and lower mortality rates [11] and the lower percentage of stroke hospitalizations resulting in death. The decrease in in-hospital case lethality rates over the last decade is likely reflecting advancements in acute stroke care [13]. The higher burden of stroke mortality has been estimated in North Asia, Eastern Europe, Central Africa, and the South Pacific [14]. In Eastern European countries, in which there are epidemics of arterial hypertension and metabolic diseases, and where there is high level of harmful risk factors for stroke, mortality from stroke has been high and without any significant changes over the past years [15, 16]. In Lithuania, time trends in stroke mortality were investigated on the basis of standardized data using criteria of the WHO MONICA Project (period 1986–2012), and a decrease in stroke mortality was reported during the study period [17].

As in many other diseases, the incidence of stroke increases dramatically with age [18, 19]. With an ageing population, despite decreasing incidence rates, the numbers of stroke events will increase as risk increases exponentially with age [20, 21].

Kaunas stroke register is the population-based register documenting all first or recurrent strokes since January 1, 1986, in the middle-aged city population [22]. The Kaunas stroke registry includes more than 300 patients each year with the first or recurrent stroke and they are evaluated including variables, such as some sociodemographic and lifestyle, and other stroke risk factors, stroke subtypes, some data from clinical investigations due to stroke, some aspects of stroke care, with long-term follow-up to 30 years [23].

The aim of this study was to evaluate trends and differences among sex and age groups in long-term survival after stroke and determine the influence of some sociodemographic and lifestyle factors, time period of stroke onset and subtype, and stroke care factors on survival.

## Material and methods

### Ethics statement

The study protocol was approved by the Lithuanian Biomedical Research Ethics Committee No 14–27. All patient data were analysed anonymously and de-identified prior to analysis.

### Study

The source of stroke cases was population-based Kaunas Stroke register, which is held between Kaunas residents aged 25–64. In this survey the population data source was the Kaunas Central Statistical Division, which annually reports on population numbers in age groups. According to the Kaunas Central Statistical Division reports in 1986–1990, 99,814 (45.3%) men and 120,508 (54.7%) women of 25–64 years of age lived on average in Kaunas, 101,398 (45.6%) men and 121,026 (54.4%) women lived in 1997–2001, and 82,747 (45.0%) and 101,020 (55.0%) men and women respectively lived in 2007–2011.

As described in detail previously [17, 22], stroke registration was performed by the recommendations of WHO MONICA project. A variety of medical information sources (hospital discharge records, outpatient department care records, necropsy, medical-legal records) were used to identify a stroke case, including death certificates from Kaunas city residents. The above indicated sources were reviewed every three months, except for death certificates that were reviewed monthly. During the study, all acute stroke events were documented using special Stroke Events Registration forms that were used in the WHO MONICA project [22]. Stroke was defined by the WHO MONICA protocol and was described in detail previously [17].

In this survey, a non-classified stroke accounted for 39 (1.0%) events. In the assessment of the data obtained, defined stroke events (fatal and non-fatal) and non-classified events (fatal and non-fatal) were used. The types of stroke were confirmed by specific diagnostic examinations and was described in detail previously [17]. In this survey, only the first-time cases of stroke were evaluated, i.e. those that happen without demonstration of a prior stroke.

The comorbidities of the stroke as main stroke risk factors, previous acute myocardial infarction (AMI), arterial hypertension (AH) and diabetes were also registered. The information about appointed rehabilitation after stroke was collected from the patient's medical records.

For the analysis of data from 1986 to 2011, three five-year periods have been taken from the Stroke Register database: 1986–1990, 1997–2001 and 2007–2011.

Survival (tracking) use for 1986–2015 death register data. Every person who suffered from stroke was traced to death, but no longer than 5 years from the stroke onset. Those who survived 5 years or more, or who died later than 5 years after stroke onset, were included in the analysis as alive (censored). Patients with no record of death were censored at December 31, 2015. Death time was confirmed by the Office for National Death Statistics.

In Kaunas city, as one of the collaborating centres of the WHO MONICA project, to keep the same methodology aspects for stroke case finding, validating and quality control throughout the study, the same stroke register group was working and the same diagnostic criteria were applied throughout the entire study period [23].

### Statistical analyses

The statistical analyses were performed with statistical software SPSS 20 version (S1 Data).

Continuous variables were summarized as mean (SD) and categorical data as percentage. One-way ANOVA was used to test differences in continuous variables where appropriate, and the χ^2^ test was used for proportions.

Survival after the onset of stroke probabilities were estimated by the Kaplan–Meier method. The Kaplan-Meier curves were plotted in order to compare 1 and 5 year survival rates between age groups, between men and women, between time periods of stroke onset and stroke types. The log-rank test was used to study differences in survival between gender and age groups, time periods of stroke onset and stroke types. The mortality of first-ever stroke rate was calculated and presented per 1,000 person-years.

Cox proportional hazard regression analysis was used to calculate gender and age specific changes in mortality over a period of time. Multivariate Cox proportional hazards (PH) models were undertaken to determine the prognostic value of some sociodemographic factors (gender and age), stroke cohorts, stroke type and some stroke risk factors (previous AMI, AH, diabetes) before stroke event. The male gender, 25–54 age group, time period of 2007–2011, ischemic stroke, no previous AMI, no history of AH, no diabetes and applied rehabilitation were used as the reference variables. The event studied was all-cause mortality. All final models were adjusted for age and gender.

In order to further assessment of the impact of rehabilitation on the risk of dying, those who did not survive 28 days were excluded from the analysis. A Cox multivariate regression model included all mentioned factors and rehabilitation variable as a time-dependent variable.

According to the scientific literature [24], for testing the PH assumption the time-dependent covariates were generated by creating interactions of the predictors and a function of survival time, and then were included in the model. If any of the time dependent covariates were significant then those predictors were not proportional. In such cases the stratified Cox regression model was used by the stratification of a covariate that does not satisfy the PH assumption. Time-dependent coefficients may be required if there are non PH in the standard Cox regression. The covariate rehabilitation (Re) violated PH assumption. Then we run the Cox PH model with rehabilitation as time-dependent variable h(t) = h_0_(t)exp(b_1_*Re + b_2_* Re * t).

Hazard ratios (HR) with 95% confidence interval (CI) for prognostic factors were calculated in Cox models. All tests were 2 tailed, and *P* value <0.05 was considered statistically significant.

## Results

Baseline characteristics of persons (men and women) aged 25–64 years, who suffered a first-ever stroke in the 3 study periods (1986–1990, 1997–2001 and 2007–2011) by age, stroke type, time period, survival duration, and prevalence of risk factors are presented in Table 1.

**Table 1:** Baseline characteristics of persons with a first-ever stroke.

Variables	All	Men	Women	P
	n (%)	n (%)	n (%)	
***No*. *of stroke events***	4129	2215	1914	
***Age group***				
25–54	1777 (43.0)	963 (43.5)	814 (42.5)	0.307
55–64	2352 (57.0)	1252 (56.5)	1100 (57.5)	0.307
***Stroke type***				
SAH	323 (7.9)	153 (7.0)	170 (9.0)	0.018
ICH	516 (12.6)	272 (12.4)	244 (12.8)	0.698
IS	3251 (79.5)	1770 (80.7)	1481 (78.2)	0.05
Unclassifiable	39 (1.0)	20 (0.9)	19 (1.0)	0.741
***Period cohorts***				
1986–1990	1298 (31.4)	746 (33.7)	552 (28.8)	0.0007
1997–2001	1465 (35.5)	721 (32.6)	744 (38.9)	0.0001
2007–2011	1366 (33.1)	748 (33.7)	618 (32.3)	0.307
***Previous AMI***	297 (7.2)	232 (10.5)	65 (3.4)	0.0001
***Arterial hypertension***	2050 (49.6)	1029 (46.5)	1021 (53.3)	0.0001
***Diabetes mellitus***	377 (9.1)	183 (8.3)	194 (10.1)	0.021

SAH-subarachnoid hemorrhage, ICH-intracerebral hemorrhage, IS-ischemic stroke, AMI-acute myocardial infarction

During 3 study periods (1986–1990, 1997–2001 and 2007–2011), 4,129 persons aged 25–64 years suffered from a first-ever stroke, of them 2,215 (53.6%) were men and 1,914 (46.4%)—women. More than a half (57.0%) of stroke patients were between the age of 55 and 64 years, while the remaining 43.0% were 25–54 years old. The proportion of men and women in these age groups was not significantly different.

Nearly four fifths of all stroke patients suffered from IS (79.5%), 12.6%—from ICH and 7.9%—from—SAH. IS was significantly more frequent in males than in females (80.7% and 78.2%, respectively, p = 0.05) and SAH was more common in women than in men (9.0% and 7.0% respectively, p = 0.018). The frequency of ICH was not significantly different between men and women during the study period (12.4% and 12.8%, respectively, p = 0.698).

All three study cohorts had a similar (about one third) proportion of all registered stroke events. In the first study cohort (1986–1990), a proportion of men suffering from stroke was significantly higher than that of women, and in the second study cohort (1997–2001), on the contrary, this proportion was significantly higher in women than in men. In the third study cohort (2007–2011), the proportions of men and women did not differ significantly.

Of all persons with first-ever stroke, 7.2% had a history of AMI: 10.5% of them were men and 3.4%—women, p = 0.0001). Almost half (49.6%) of the subjects had history of AH (more women (53.3%) than men (46.5%, p = 0.0001)). More than 9% of stroke patients had history of DM (10.1% women and 8.3% men, p = 0.021).

Survival and mortality data among investigated stroke events with a first-ever stroke by sex, survival time and study period are presented in Table 2.

**Table 2:** Survival and mortality data among persons with a first-ever stroke by sex, survival time and study period.

Variables	All	Men	Women	P
	n (%)	n (%)	n (%)	
***Survival***				
1 year	3272 (79.2)	1704 (76.9)	1568 (81.9)	0.0001
5 year	2905 (70.4)	1452 (65.6)	1453 (75.9)	0.0001
***Mortality per 1*,*000 person-years***				
***1 year***				
1986–1990	371.74	385.18	353.78	0.15
1997–2001	232.01	283.81	185.07	0.00001
2007–2011	182.85	212.09	149.09	0.00002
***5 years***				
1986–1990	125.84	138.69	109.34	0.046
1997–2001	71.68	91.98	54.01	0.001
2007–2011	47.53	60.22	33.28	0.004

SAH-subarachnoid hemorrhage, ICH-intracerebral hemorrhage, IS-ischemic stroke

Of 4,129 persons with a first-ever stroke, 3,272 (79.2%) persons survived 1 year and 2,905 (70.4%) persons survived 5 years after stroke onset. Among men, 1-year survival rate was significantly lower than that among women (76.9% and 81.9%, respectively, p = 0.0001). Comparing 5-year survival among persons with first-ever stroke by gender, similar differences in survival rates were determined. The proportions of persons who survived 1 and 5 years after stroke also were highest in persons with IS (87.1% and 57.0%, respectively) and the lowest ones were in persons who suffered from a SAH (76.8% and 53.9%, respectively) (p = 0.0001).

Both 1 and 5 year first-ever stroke survival rate was higher among persons who suffered their stroke in 2007–2011 (84.3% and 72.2%, respectively) than among those who suffered their stroke in 1986–1990 (80.2% and 57.9%, respectively, p = 0.0001). Survival in cohort of persons, who suffered stroke in 1997–2001, was significantly higher than that in cohort from 1986–1990.

Assessing one and five-year mortality rates per 1,000 person-years among men and women in 3 cohorts, it was found that male mortality was about 2-fold higher than for women, except for 1-year mortality cohort in 1986–1990. When assessing 1-year and 5-year mortality per 1,000 person-years in 3 cohorts, it was estimated that in the last cohort (2007–2011) the mortality rates for both men and women were lower compared with the first cohort (1986–1990); for 1 year mortality were on average 1.8 and 2.4 times and for 5 year mortality were on average 2.3 and 3.3 times lower, respectively (Table 2).

**Fig 1:**
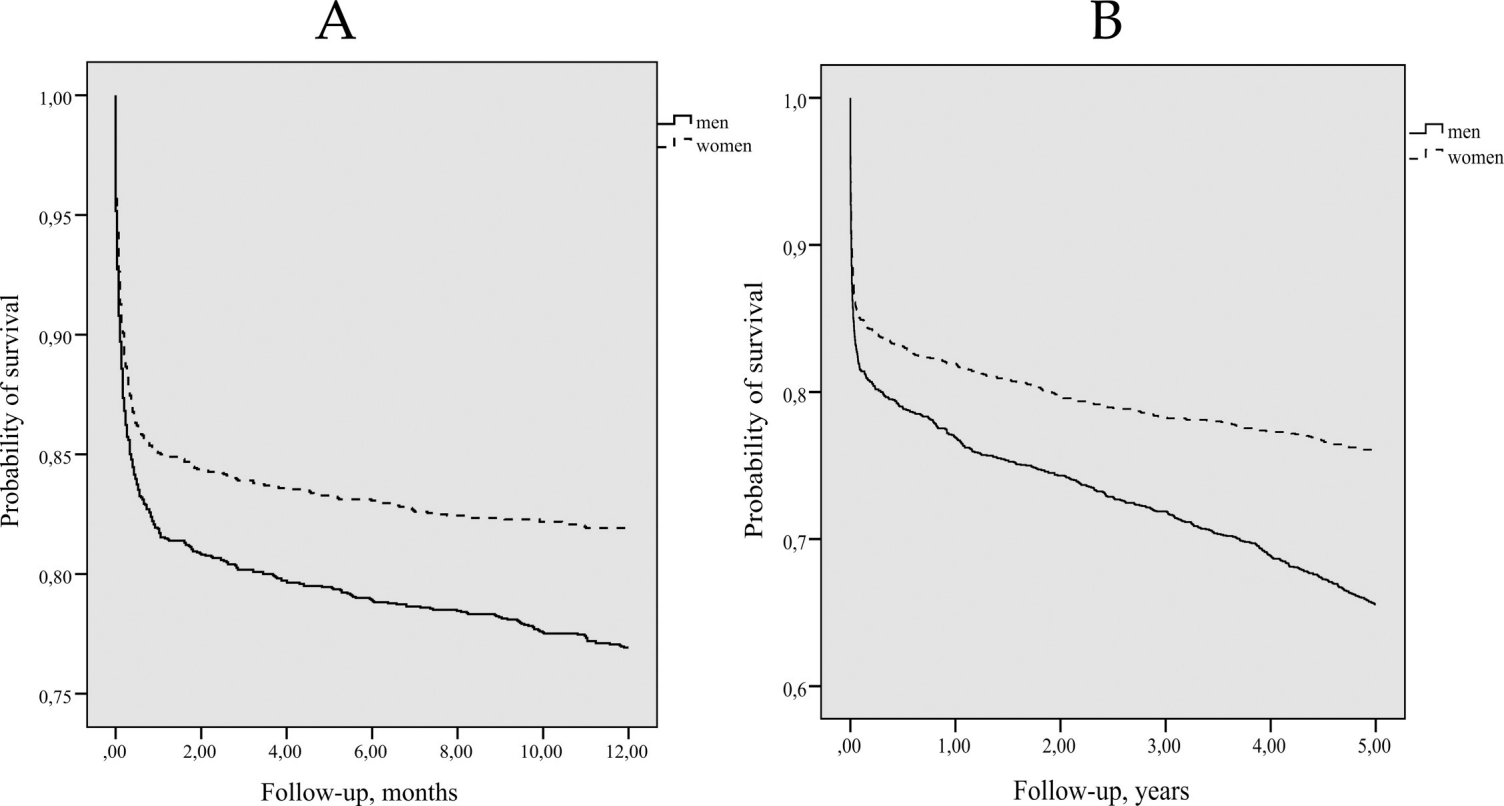
Kaplan-Meier 1-year survival (A) and 5-years survival (B) curves for persons with first-ever stroke by gender. *(A) Log-rank = 14*.*922*, *p = 0*.*0001*. *(B) Log-rank = 47*.*657*, *p = 0*.*0001*.

**Fig 2:**
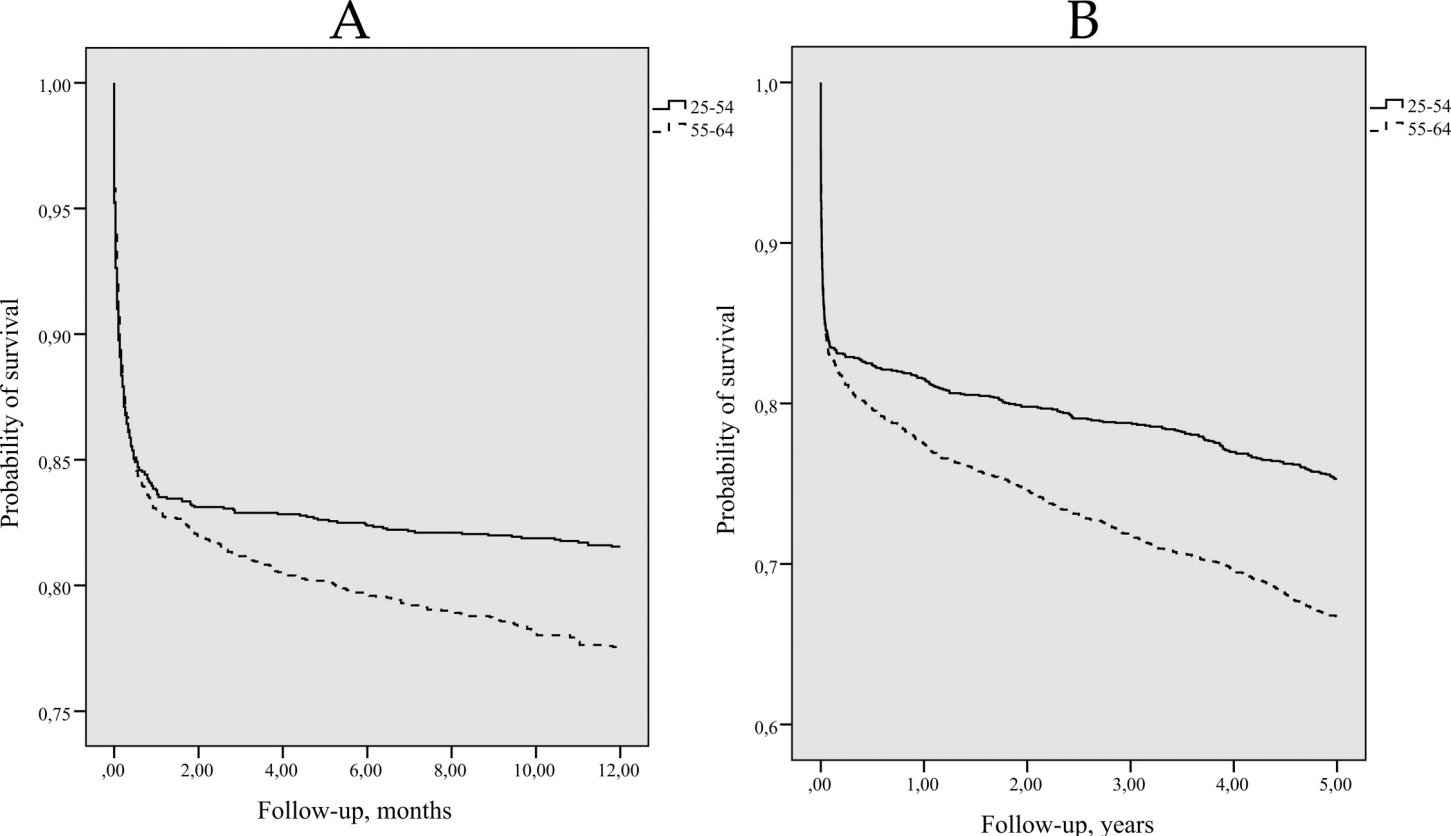
Kaplan-Meier 1-year survival (A) and 5-years survival (B) curves for persons with first-ever stroke by age. *(A) Log-rank = 8*.*243*, *p = 0*.*004*. *(B) Log-rank = 31*.*06*, *p = 0*.*0001*.

Kaplan-Meier long-term survival curves for persons with first-ever stroke by gender are presented in Fig 1A and Fig 1B and those by age group–in Fig 2A and Fig 2B. The majority (83.4%) of stroke patients survived within the first 28 days after the onset of the stroke. The survival in women during 1- and 5-years after a first-ever stroke was significantly better compared with men (Log-rank test p = 0.0001).

The older (55–64 year) first-ever stroke patients had a lower 1-year and 5-years probability of survival than those of younger age (25–54 years) (Log-rank test p = 0.0001), but during first 28 days of stroke onset surviving among 25–54 and 55–64 age groups patients did not differ significantly, 83.8% and 83.1% respectively.

**Fig 3:**
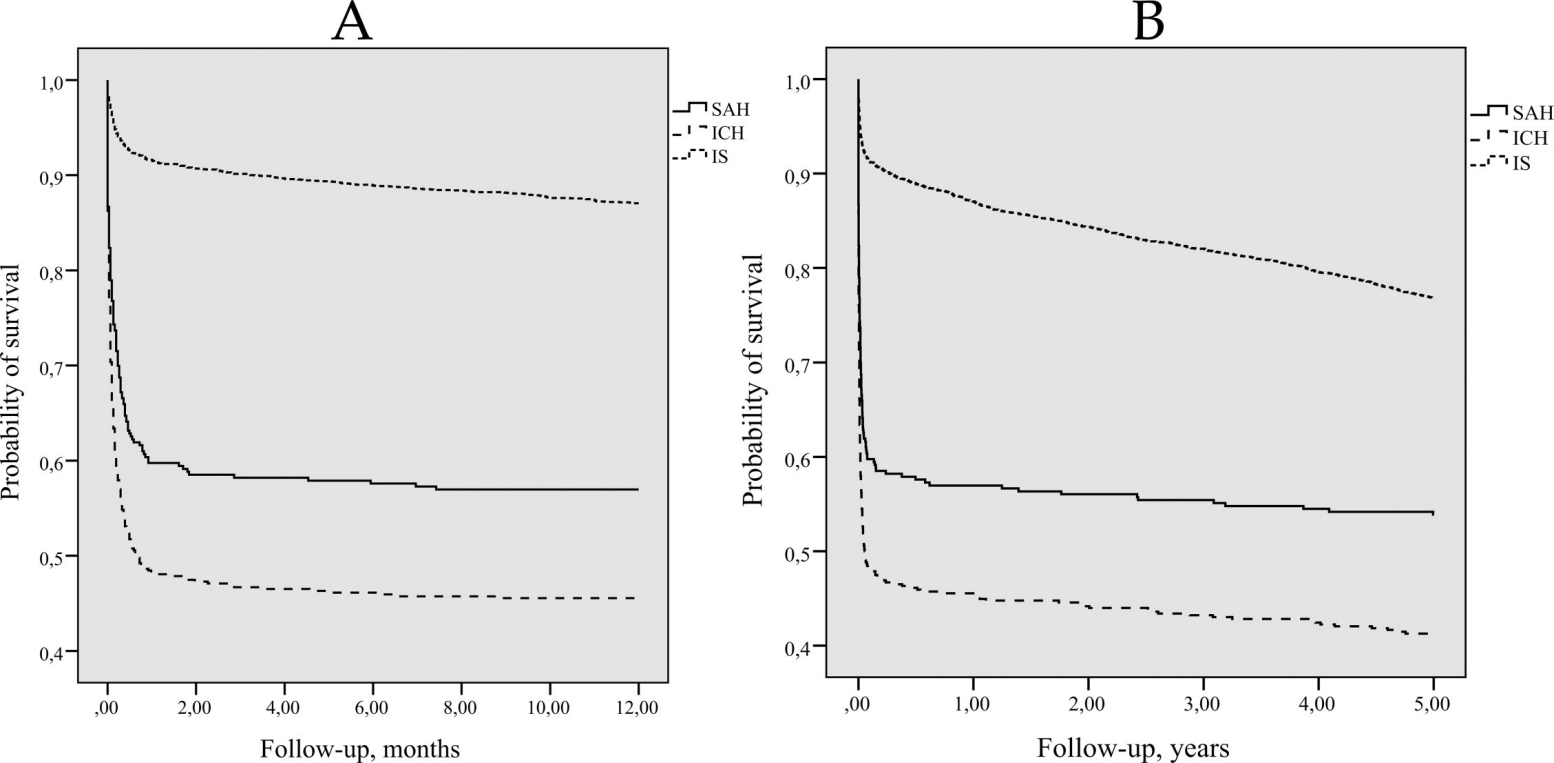
Kaplan-Meier 1-year survival (A) and 5-years survival (B) curves for persons with first-ever stroke by type. *(A) Log-rank = 10*.*927*, *p = 0*.*001; comparing SAH and ICH (B) Log-rank = 13*.*187*, *p = 0*.*0001; comparing SAH and ICH*. *(A) Log-rank = 265*.*501*, *p = 0*.*0001; comparing SAH and IS (B) Log-rank = 133*.*620*, *p = 0*.*0001; comparing SAH and IS*. *(A) Log-rank = 709*.*62*, *p = 0*.*0001; comparing ICH and IS (B) Log-rank = 482*.*389*, *p = 0*.*0001; comparing ICH and IS*.

The persons with the first-ever IS had a better chance to survive first 1- and 5-years after stroke compared with persons who had ICH or SAH (Log-rank test p = 0.0001) (Fig 3A and Fig 3B). Among persons with first-ever SAH, 1- and 5-year survival rates were higher compared to 1- and 5-year survival rates of persons who suffered ICH (Log-rank test p = 0.0001).

**Fig 4:**
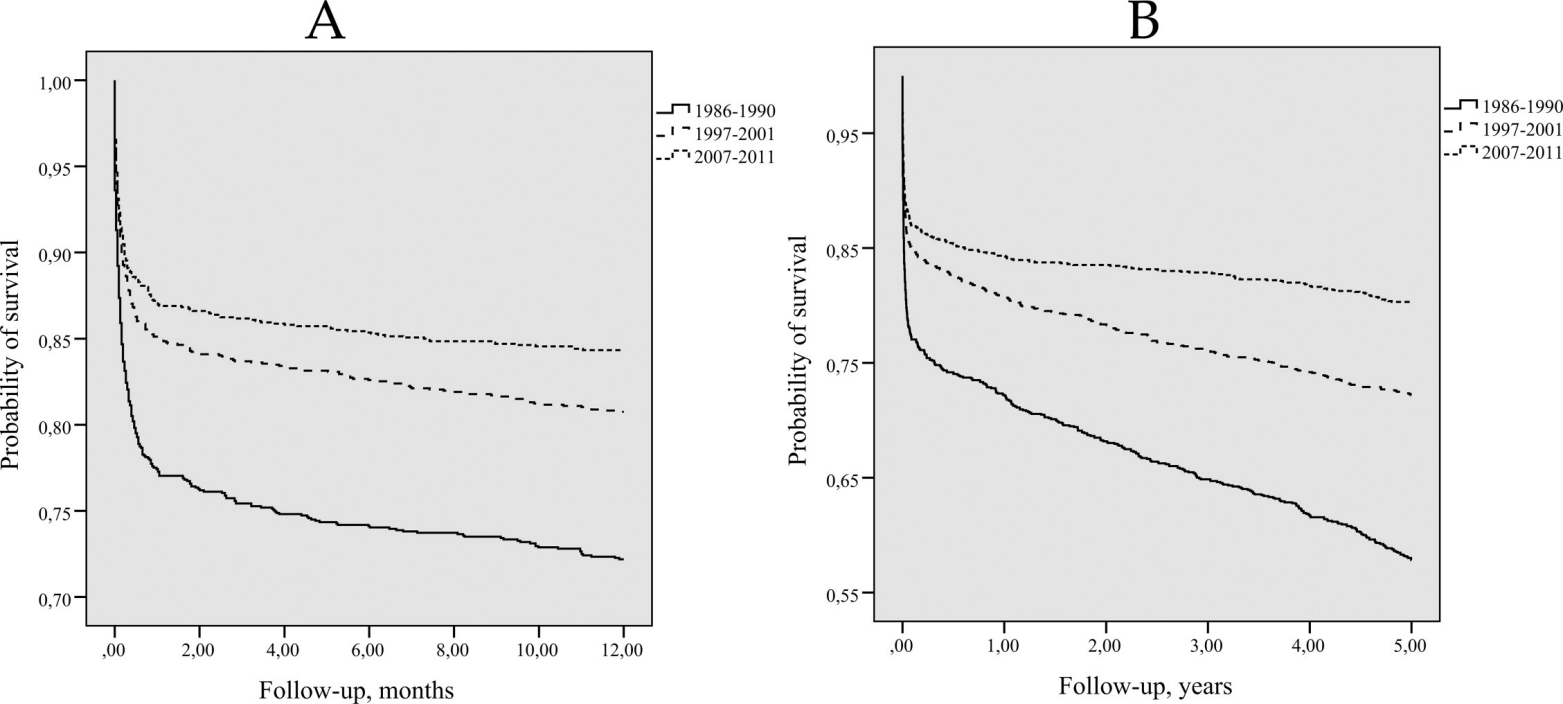
Kaplan-Meier 1-year survival (A) and 5-years survival (B) curves for persons with first-ever stroke by time cohort. *(A) Log-rank = 28*.*836*, *p = 0*.*0001; comparing 1986–1990 (B) Log-rank = 61*.*082*, *p = 0*.*0001; comparing 1986–1990 and 1997–2001 and 1997–2001*.*(A) Log-rank = 57*.*466*, *p = 0*.*0001; comparing 1986–1990 (B) Log-rank = 148*.*873*, *p = 0*.*0001; comparing 1986–1990 and 2007–2011 and 2007–2011*.*(A) Log-rank = 5*.*969*, *p = 0*.*015; comparing 1997–2001 (B) Log-rank = 22*.*935*, *p = 0*.*0001; comparing 1997–2001 and 2007–2011 and 2007–2011*.

In addition, long-term first-ever stroke survival rates by time cohort were evaluated (Fig 4A and Fig 4B). Among persons who had had a stroke attack in 2007–2011, 1- and 5-year survival rates were higher compared to those in persons who had suffered from a stroke in 1986–1990 or in 1997–2001 (Log-rank test p = 0.0001). In addition, 1 and 5 year survival rates were higher among persons who had had their first-ever stroke in 1997–2001, compared to the persons who had had a stroke in 1986–1990 (Log-rank test p = 0.0001).

Kaplan-Meier 1-year survival curves for persons suffered SAH, ICH and IS by gender (S1 Fig), by age (S2 Fig) and by time cohort (S3 Fig) are presented as supporting information. Kaplan-Meier 5-years survival curves for persons suffered SAH, ICH and IS by gender (S4 Fig), by age (S5 Fig) and by time cohort (S6 Fig) also are presented as supporting information. According to 1- and 5-year survival data, female and younger age group survival significantly was better only in IS group. Assessing the survival rates of 1 and 5 years in time cohorts, it was found that these survival rates for both SAH, ICH and IS were significantly better among individuals with the above-mentioned stroke types during the 2007–2011 period.

Factors predicting death from any cause during 1 year among persons with a first-ever stroke who survived first 28 days by stroke types are presented in Table 3. Multivariate survival analyses showed that female gender (HR = 0.52, 95% CI 0.36–0.74) was associated with higher 1-year survival rates after the first-ever stroke only among IS cases. On the other hand, older age (HR = 2.54, 95% CI 1.69–3.82), history of previous AMI or DM (HR = 1.73, 95% CI 1.11–2.7 and HR = 1.87, 95% CI 1.21–2.88 respectively) and not using rehabilitation (HR = 2.65, 95% CI 1.32–5.33) were associated with poorer 1-year survival after the first-ever stroke only among IS cases too. For ICH stroke cases only the second investigated time cohort (1997–2001) were associated with poorer 1-year survival after the first-ever stroke (HR = 7.4, 95% CI 1.59–34.4) as compared with last time cohort (2007–2011). Among SAH stroke events any of investigated variables were not associated with better or worse 1-year survival after a first-ever stroke (Table 3).

**Table 3:** All-cause mortality risk during 1 year for 25 to 64 years aged persons with the first-ever stroke by stroke types (n = 3420) (Cox regression analysis).

Variables	Stroke types					
	SAH		ICH		IS	
	HR	95% CI	HR	95% CI	HR	95% CI
**Gender (Men ref.)**	1.3	0.33–5.07	2.45	0.84–7.34	**0.52**	**0.36–0.74**
**Age group (25–54 ref.)**	0.86	0.21–3.6	1.73	0.57–5.25	**2.54**	**1.69–3.82**
**Time periods:**						
**2007–2011 (ref.)**	1		1		1	
**1986–1990**	0.51	0.7–3.46	6.71	0.84–53.5	0.98	0.54–1.75
**1997–2001**	0.35	0.07–1.81	**7.4**	**1.59–34.4**	1.2	0.74–1.94
**Previous AMI (No ref.)**	0^1^	0^1^	0^1^	0^1^	**1.73**	**1.11–2.7**
**Arterial hypertension (No ref.)**	1.5	0.39–5.84	0.38	0.13–1.11	0.78	0.54–1.12
**Diabetes mellitus (No ref.)**	0^1^	0^1^	2.84	0.59–13.68	**1.87**	**1.21–2.88**
**Rehabilitation (Yes ref.)**^2^	9.04	0.34–241.0	0.52	0.06–4.79	**2.65**	**1.32–5.33**
Rehabilitation*time	1.44	0.76–2.78	0.99	0.6–1.63	**1.02**	**0.93–1.13**

HR–hazard risk, 95% CI–confidence interval, SAH-subarachnoid hemorrhage, ICH-intracerebral hemorrhage, IS-ischemic stroke, AMI-acute myocardial infarction

^1^ because of the small number of cases, there is not one event (death) with the „previous AMI”or with „diabetes mellitus“

^2^ included in model as „time-depended variable“

Rehabilitation*time—rehabilitation multiplied by time.

Similar predicting variables as for 1-year survival, were determined among persons after first-ever stroke when 5-year survival was analysed (Table 4). Survival was significantly predicted by gender, age, previous AMI, DM and rehabilitation after stroke event. Among IS cases 5-years survival were poorer among men (HR = 0.5, 95% CI 0.41–0.61), in 1986–1990 and 1997–2001 times cohorts as compared with 2007–2011 (HR = 2.68, 95% CI 1.91–3.76 and HR = 2.03, 95% CI 1.53–2.69 respectively), among persons with previous AMI and DM (HR = 1.79, 95% CI 1.39–2.31 and HR = 2.05, 95% CI 1.61–2.61) and in persons not using rehabilitation (HR = 1.56, 95% CI 1.09–2.23). For ICH stroke cases only oldest age (HR = 2.06, 95% CI 1.01–4.21) and the first two studied time cohorts (1986–1990 and 1997–2001) were associated with poorer 5-year survival after the first-ever stroke (HR = 7.54, 95% CI 2.46–23.13 and HR = 2.29, 95% CI 1.02–5.14 respectively) as compared with last time cohort (2007–2011). Among SAH stroke events any of investigated variables were not associated with better or worse 5-year survival after a first-ever stroke (Table 4).

**Table 4:** All-cause mortality risk during 5 years for 25 to 64 years aged persons with the first-ever stroke by stroke types (n = 3420) (Cox regression analysis).

Variables	Stroke types					
	SAH		ICH^3^		IS^4^	
	HR	95% CI	HR	95% CI	HR	95% CI
**Gender (Men ref.)**	0.79	0.31–2.01	-	-	**0.5**	**0.41–0.61**
**Age group (25–54 ref.)**	0.96	0.36–2.59	**2.06**	**1.01–4.21**	**-**	**-**
**Time periods:**						
**2007–2011 (ref.)**	1		1		1	
**1986–1990**	2	0.52–7.7	**7.54**	**2.46–23.13**	**2.68**	**1.91–3.76**
**1997–2001**	0.62	0.17–2.67	**2.29**	**1.02–5.14**	**2.03**	**1.53–2.69**
**Previous AMI (No ref.)**	0^1^	0^1^	0.8	0.1–6.42	**1.79**	**1.39–2.31**
**Arterial hypertension (No ref.)**	1.02	0.38–2.77	1.41	0.60–3.33	1.08	0.88–1.31
**Diabetes mellitus (No ref.)**	0^1^	0^1^	2.52	0.92–6.93	**2.05**	**1.61–2.61**
**Rehabilitation (Yes ref.)**^2^	0.99	0.20–4.82	0.57	0.16–1.97	**1.56**	**1.09–2.23**
Rehabilitation*time	0.77	0.36–1.61	0.99	0.66–1.49	**0.99**	**0.89–1.12**

HR–hazard risk, 95% CI–confidence interval, SAH-subarachnoid hemorrhage, ICH-intracerebral hemorrhage, IS-ischemic stroke, AMI-acute myocardial infarction

^1^ because of the small number of cases, there is not one event (death) with the „previous AMI”or with „diabetes mellitus“

^2^ included in model as „time-depended variable“

^3^ variable “gender" is included in the model as a stratum, since it did not meet the assumptions of the proportionality of risks

^4^ variable "age group" is included in the model as a stratum, since it did not meet the assumptions of the proportionality of risks

Rehabilitation*time—rehabilitation multiplied by time.

## Discussion

We conducted a population-based study of 1- and 5-year survival after stroke in a large defined urban population in one of the Eastern European countries—Lithuania. Our study provides prognostic data from a large, unselected, population-based cohort of patients with stroke diagnosed retrospectively according to MONICA study protocol and using standardized diagnostic criteria. The outcome events were carefully defined.

Looking at long-term survival rates, it was found that almost 80% of middle-aged persons with a first-ever stroke survived for 1 year, and those who survived 5 years were as many as 70%. Similar data were also provided by other researchers who assessed post stroke survival in some European countries. In Spain (Barcelona stroke registry) in-hospital survival after stroke from 1986–1992 years period to 2006–2009 years period significantly increased from 82.4% to 89.0% respectively [8]. By the data from Riks Stroke Database (Sweden) 1 year survival after ischemic stroke was 78% and after hemorrhagic stroke only 62% [9]. In some European countries, as England, Belarus, it was worse than in Lithuania. Data from South London Stroke Register revieled that 1 year survival after stroke was 63.7% and 5 year survival after stroke only 42.8%, although the average age of the subjects in this study was higher than the mean age of the subjects in our study [25]. According to data in GROSS Stroke Study (Belarus) among persons under 65 years 1 year survival after a first-ever stroke was 72% and after 5 years after stroke– 58% [15]. Increasing stroke survival rates among persons with stroke has recently been reported in the United Kingdom [26], Estonia [27], France [28], Netherlands [29] and others Western European countries [30].

In our study, we found that proportions of women who survived 1 and 5 years after the stroke were significantly higher than in men. This may be due to the fact that the prevalence of harmful lifestyle risk factors in Lithuanian men is significantly higher than in women [31]. Men are less likely to control their blood pressure effectively, and among men other chronic non-communicable diseases such as ischemic heart disease, DM, which can increase the chance of death, are diagnosed more frequently [9, 32]. Additionally, harmful risk factors such as smoking, alcohol usage prevalence among Lithuanian men are higher than among women [31]. On the other hand, among our investigated persons with stroke the prevalence of history of AH and DM was significantly higher among women than men.

We demonstrated that there was a significant difference in survival rates by type of index stroke during 1 and 5 years period. Less than half of those with an intracerebral hemorrhage survived for 1 or 5 years. Of those with SAH 1 year survival rate was 57% and that of 5 years—54%. The vast majority of the patients who suffered IS (around 90%), survived 1 year after the stroke and 77% survived 5 years. In some studies, significantly lower numbers of people with hemorrhagic stroke experienced complications which were more lethal and might have led to a higher mortality rate in these patients [33, 34]. It is important to mention that the long-term survival rates of Kaunas middle-aged people in case of IS were similar to those in England and Sweden [25, 35]. This allows us to expect that health care and follow-up of these individuals in future could improve survival rates by successfully controlling levels of most common risk factors for stroke, reducing the incidence of life-threatening risk factors, successfully treating other chronic non-communicable diseases, which potentially exacerbate stroke.

In assessing survival rates in study cohorts, it was found that the best survival rates for both 1 and 5 years in stroke were in the cohort of 2007–2011 compared with previous cohorts in both 1986–1990 and 1997–2001. This suggests that in the last stroke cohort patients were treated most intensively. Urgent blood pressure lowering was recorded in 67% of persons in Kaunas, but in some European countries it’s amounted about 40% in overall [36]. Of the evidence-based, cost effective drugs overall, in Kaunas 74% of patients were appropriately prescribed an antiplatelet during the hospital admission, as like that in the other European cities as Dijon, London, Warszaw. The use of anticoagulants and its impact on survival after stroke especially in stroke patients with ischemic stroke was of particular interest. By the 2004–2007 years data of Kaunas stroke register the frequency of anticoagulants using among stroke patients was 18.3% and was 2 times frequently for stroke patients with atrial fibrillation (37.3%) [36]. In addition, thrombolysis as stroke treatment method was introduced in the last stroke cohort, which was able to improve the survival rates in cases of stroke, however by data of Kaunas stroke register in period from 2004 to 2007 years thrombolysis was not performed [36].

As commonly acknowledged, the increase in stroke survival could be explained by improvements in stroke management. In Kaunas, every fourth person with stroke was admitted to the stroke unit, where one in five spent more than 50% of time. By the data from 2004 to 2007, in Kaunas more than 95% of stroke patients were seen by stroke physician which could lead fact that these patients becoming more likely to fall into hospital stroke unit [36]. In addition, improved management in specialized stroke care units and secondary prevention could lead to better survival, especially among those aged <65 years [35].

The level and controlling of main risk factors for stroke, such as AH and high blood glucose level, has also improved, therefore the frequency of harmful behaviour has decreased among Kaunas middle-aged population in the last years [31]. During the past 10 years, among Kaunas inhabitants AH awareness among hypertensive persons increased to 64.4% in men and to 72.3% in women [37].

Recent treatments, including thrombectomy, are currently available to patients with stroke in Lithuania, which could lead to lower mortality in the future among stroke patients.

According to Swedish Inpatient Register the long-term survival improved over the past twenty years in both men and women and the mortality risk over that time decreased by one third in men and nearly fifty percent in women [38]. An increase in stroke survival in Sweden could be explained by perfection in treatment and positive cardiovascular risk factors transformation over the past decades.

During the study period in Sweden, the prevalence of AH and smoking have decreased [39]. Should be noted, that lower prevalence of AH could be interpreted by certain dietary modifications and higher quality antihypertensive treatment for the hypertensive persons, while lower smoking prevalence could be partly explained by better knowledge of people and by strong intervention programs among smoking peoples [40, 41]. In recent years, treatment and diabetes control in diabetic patients has improved in some countries [42].

By assessing factors that had a significant relationship with a worse 1-year and 5-year survival, our study showed that male gender, older age, history of AMI, DM, the first studied time cohorts (1986–1990 and 1997–2001) and lack of rehabilitation significantly increased the likelihood of death, i.e. worse survival among IS cases and older age and the first studied time cohorts (1986–1990 and 1997–2001) increased the likelihood of death in ICH cases. The data from prospective observational study of 1,030 stroke patients in four regions of Italy showed that predictors of increased mortality rates over six months controlling data by demographic factors, pre-stroke functions, underlying illnesses and risk factors were altered consciousness, hyperthermia, pneumonia, and heart failure, while antiplatelet therapy and early mobilisation significantly increased survival during 6 months after stroke [43]. Among SAH stroke events any of investigated variables were not associated with better or worse 1- and 5-year survival after stroke. As unchanged, arterial hypertension, like a stroke risk factor, did not significantly increase the likelihood of death in one and five years. This may be due to the fact that people who had a stroke controlled their blood pressure sufficiently well when using antihypertensive drugs [35]. According to other researchers, AH was also not a predictor of death for 6 months after the stroke that increased the chance of death for both total and ischemic stroke, although the frequency of AH among the dead within 6 months post-stroke subjects was significantly higher compared to survivors [43]. Similar results are confirmed by other researchers. According to univariate regression analysis, the AH, diabetes and smoking among Chinese population were not associated with significantly higher mortality over a 3-month period after stroke [44].

These and a number of other factors that increased the likelihood of death were also reported by a number of other researchers who assessed the long-term survival of stroke [10, 44, 45]. It is interesting to note that the type of stroke did not lead to a worse 1 and 5 year survival for those who survived the first 28 days after the stroke, although having assessed all cases of stroke, we noticed that the hemorrhagic and subarachnoid type of stroke significantly led to higher mortality. It can be argued that long-term survival was more influenced not by clinical signs or complications, but by chronic concomitant illness, bad control of stroke risk factors or harmful lifestyle [43, 46, 47].

It is worth noting that the assessment of mortality in a stroke can also be related to methodological problems in evaluating various predictors of mortality from stroke. According to the meta-analysis data, the described 66 different prognostic models had poor methodological justification, and less than half of all models used in mortality after IS had only external validation. In the future required other external validation and model impact studies to confirm the usefulness of existing prognostic models depending of their context for decision making [48].

Therefore, to further reduce the mortality and improve the long-term survival among stroke patients, more intensive and extended secondary interventions are warranted, including both medication, rehabilitation, control of stroke risk factors and lifestyle changes.

### Limitations

There are some limitations as well as several strengths to our study. Strengths include a sufficiently large, representative population, a sufficiently long time of stroke cases collection, and use of population-based register data. There are several limitations in our study. We do not have some lipid metabolism indicators in blood as total cholesterol levels data in our stroke registry, as well as levels of inflammation indicators and some neuroendocrine biomarkers in blood. Also we did not have any data on the treatment provided and procedures performed in hospital and after leaving the hospital after stroke. Also, there were no possibilities to get some clinical data for stroke patients such as diagnosis of other cardiovascular diseases (atrial fibrillation), chronic kidney diseases, loss function level, and data of function recovery. In our study we were also unable to evaluate the type, number of sessions, starting period, duration of rehabilitation, because our stroke register not collected these data. Moreover, we did not have personal data on their lifestyle risk factors, such as smoking and alcohol consumption, which could have had an effect on survival after stroke. Besides, we did not have data about nutrition peculiarities after stroke. Also, we did not have any data about the work of people after stroke as well as the data on their home and work environment in which the person lived.

## Conclusions

This population-based study of patients with stroke found that the long-term survival was better in women than men and improved significantly in both men and women during the past years. Long-term survival was better in the younger age and among the stroke survivors who have had IS. Possible explanations for this trend could be attributed to better secondary prevention, improved treatment of stroke risk factors, and the rehabilitation procedures. Probability of long-term survival was increased due to women gender, younger age, IS type, absence of previous AMI and DM history. The risk of mortality among stroke survivors varies by age and stroke type groups, and further work should attempt to identify factors in these groups. Our data on the long-term stroke outcomes are relevant for public health prevention programs, as well as for the evidence based rehabilitation service planning and health supplies distribution.

## Supporting information

S1 Fig **Kaplan-Meier 1-year survival curves for persons with subarachnoidal hemorraghe (A), ischemic cerebral hemorraghe (B) and ischemic stroke (C) by gender**.
(A) Log-rank = 0.004, p = 0.952, (B) Log-rank = 0.070, p = 0.791, (C) Log-rank = 38.901, p = 0.0001
(TIF)

S2 Fig **Kaplan-Meier 1-year survival curves for persons with subarachnoidal hemorraghe (A), ischemic cerebral hemorraghe (B) and ischemic stroke (C) by age**.
(A) Log-rank = 2.432, p = 0.119, (B) Log-rank = 0.016, p = 0.899, (C) Log-rank = 40.521, p = 0.0001
(TIF)

S3 Fig **Kaplan-Meier 1-year survival curves for persons with subarachnoidal hemorraghe (A), ischemic cerebral hemorraghe (B) and ischemic stroke (C) by time cohort**.
(A) Log-rank = 14.30, p = 0.0001; comparing 1986–1990 with 1997–2001,
(A) Log-rank = 8.216, p = 0.004; comparing 1986–1990 with 2007–2011
(A) Log-rank = 0.389, p = 0.533; comparing 1997–2001 with 2007–2011
(B) Log-rank = 19.541, p = 0.0001; comparing 1986–1990 with 1997–2001,
(B) Log-rank = 31.277, p = 0.0001; comparing 1986–1990 with 2007–2011
(B) Log-rank = 2.942, p = 0.086; comparing 1997–2001 with 2007–2011
(C) Log-rank = 22.407, p = 0.0001; comparing 1986–1990 with 1997–2001,
(C) Log-rank = 43.978, p = 0.0001; comparing 1986–1990 with 2007–2011
(C) Log-rank = 4.314, p = 0.038; comparing 1997–2001 with 2007–2011
(TIF)

S4 Fig **Kaplan-Meier 5-year survival curves for persons with subarachnoidal hemorraghe (A), ischemic cerebral hemorraghe (B) and ischemic stroke (C) by gender**.
(A) Log-rank = 0.025, p = 0.874, (B) Log-rank = 0.716, p = 0.397, (C) Log-rank = 77.153, p = 0.0001
(TIF)

S5 Fig **Kaplan-Meier 5-year survival curves for persons with subarachnoidal hemorraghe (A), ischemic cerebral hemorraghe (B) and ischemic stroke (C) by age**.
(A) Log-rank = 2.015, p = 0.156, (B) Log-rank = 0.111, p = 0.739, (C) Log-rank = 69.384, p = 0.0001
(TIF)

S6 Fig **Kaplan-Meier 5-year survival curves for persons with subarachnoidal hemorraghe (A), ischemic cerebral hemorraghe (B) and ischemic stroke (C) by time cohort**.
(A) Log-rank = 19.133, p = 0.0001; comparing 1986–1990 with 1997–2001,
(A) Log-rank = 11.828, p = 0.001; comparing 1986–1990 with 2007–2011
(A) Log-rank = 0.260, p = 0.610; comparing 1997–2001 with 2007–2011
(B) Log-rank = 24.050, p = 0.0001; comparing 1986–1990 with 1997–2001,
(B) Log-rank = 36.808, p = 0.0001; comparing 1986–1990 with 2007–2011
(B) Log-rank = 2.677, p = 0.102; comparing 1997–2001 with 2007–2011
(C) Log-rank = 46.546, p = 0.0001; comparing 1986–1990 with 1997–2001,
(C) Log-rank = 133.720, p = 0.0001; comparing 1986–1990 with 2007–2011
(C) Log-rank = 26.098, p = 0.0001; comparing 1997–2001 with 2007–2011
(TIF)

S1 Data In the file „Data_mini_set.xls”are presented crude data, from which were calculated and evaluated data included in the manuscript. (XLS)
