# Supplementary figures and images for: Long-term survival after stroke in Lithuania: Data from Kaunas population-based stroke registry
Source: PLoS One. 2019 Jul 10;14(7):e0219392. doi: 10.1371/journal.pone.0219392 (PMC6619798; doi:10.1371/journal.pone.0219392)

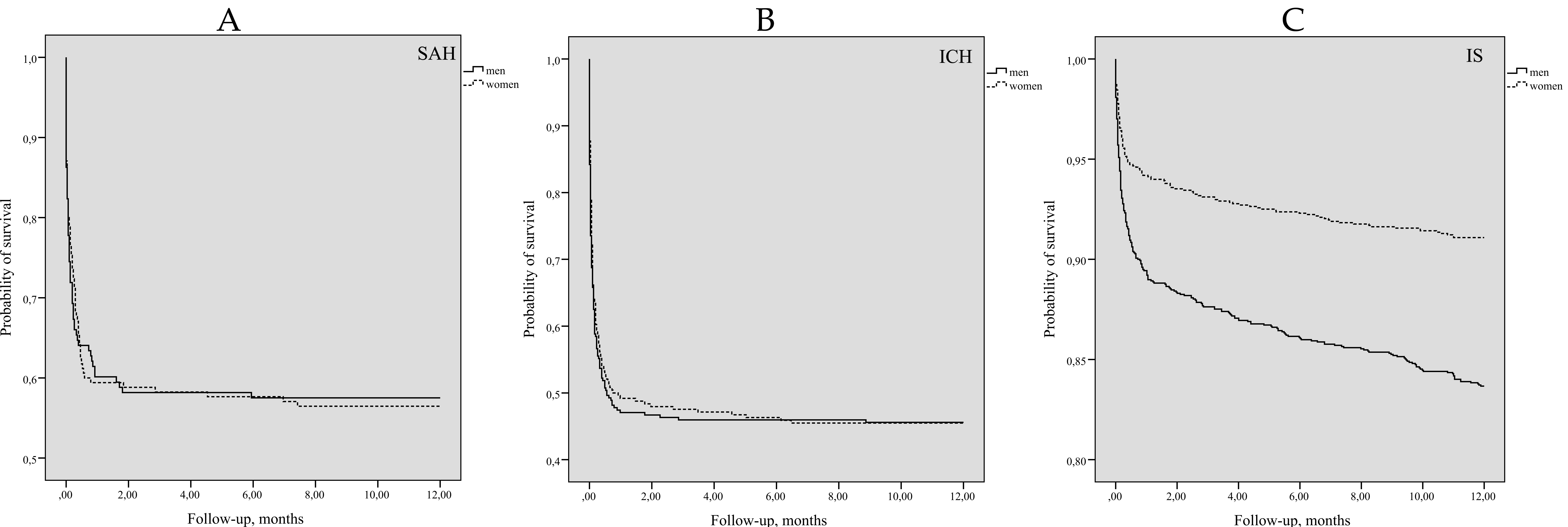

Supplement: S1 Fig — Kaplan-Meier 1-year survival curves for persons with subarachnoidal hemorraghe (A), ischemic cerebral hemorraghe (B) and ischemic stroke (C) by gender. (A) Log-rank = 0.004, p = 0.952, (B) Log-rank = 0.070, p = 0.791, (C) Log-rank = 38.901, p = 0.0001 (TIF) [file pone.0219392.s001.tif]

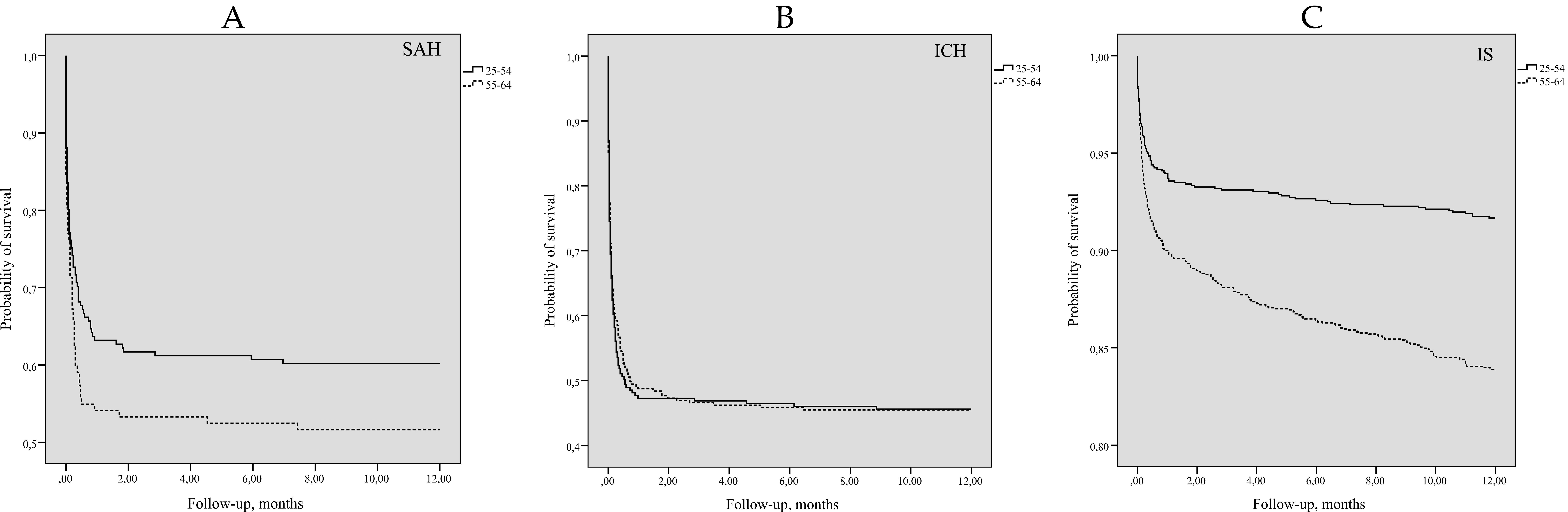

Supplement: S2 Fig — Kaplan-Meier 1-year survival curves for persons with subarachnoidal hemorraghe (A), ischemic cerebral hemorraghe (B) and ischemic stroke (C) by age. (A) Log-rank = 2.432, p = 0.119, (B) Log-rank = 0.016, p = 0.899, (C) Log-rank = 40.521, p = 0.0001 (TIF) [file pone.0219392.s002.tif]

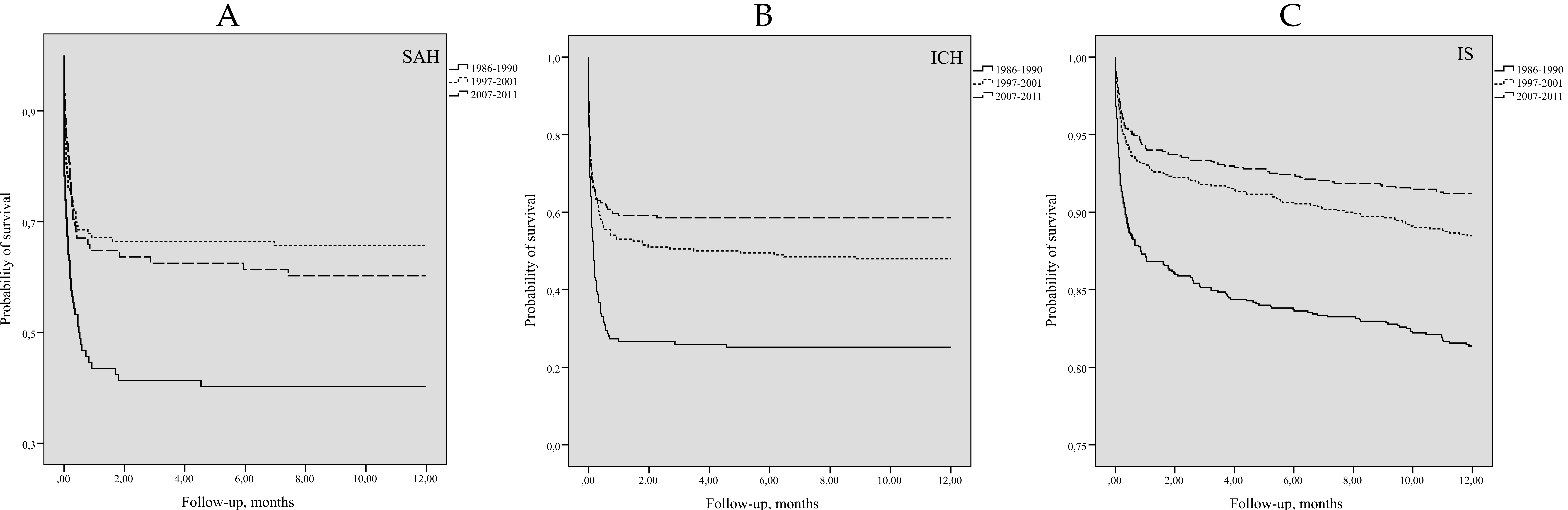

Supplement: S3 Fig — Kaplan-Meier 1-year survival curves for persons with subarachnoidal hemorraghe (A), ischemic cerebral hemorraghe (B) and ischemic stroke (C) by time cohort. (A) Log-rank = 14.30, p = 0.0001; comparing 1986–1990 with 1997–2001, (A) Log-rank = 8.216, p = 0.004; comparing 1986–1990 with 2007–2011 (A) Log-rank = 0.389, p = 0.533; comparing 1997–2001 with 2007–2011 (B) Log-rank = 19.541, p = 0.0001; comparing 1986–1990 with 1997–2001, (B) Log-rank = 31.277, p = 0.0001; comparing 1986–1990 with 2007–2011 (B) Log-rank = 2.942, p = 0.086; comparing 1997–2001 with 2007–2011 (C) Log-rank = 22.407, p = 0.0001; comparing 1986–1990 with 1997–2001, (C) Log-rank = 43.978, p = 0.0001; comparing 1986–1990 with 2007–2011 (C) Log-rank = 4.314, p = 0.038; comparing 1997–2001 with 2007–2011 (TIF) [file pone.0219392.s003.tif]

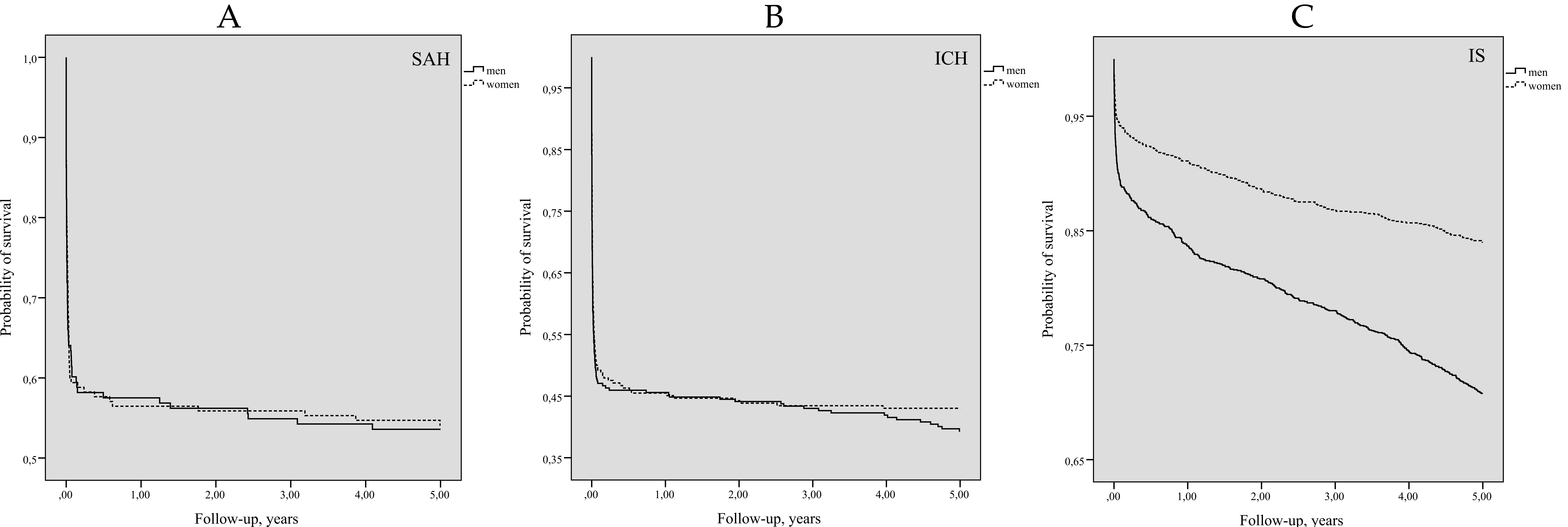

Supplement: S4 Fig — Kaplan-Meier 5-year survival curves for persons with subarachnoidal hemorraghe (A), ischemic cerebral hemorraghe (B) and ischemic stroke (C) by gender. (A) Log-rank = 0.025, p = 0.874, (B) Log-rank = 0.716, p = 0.397, (C) Log-rank = 77.153, p = 0.0001 (TIF) [file pone.0219392.s004.tif]

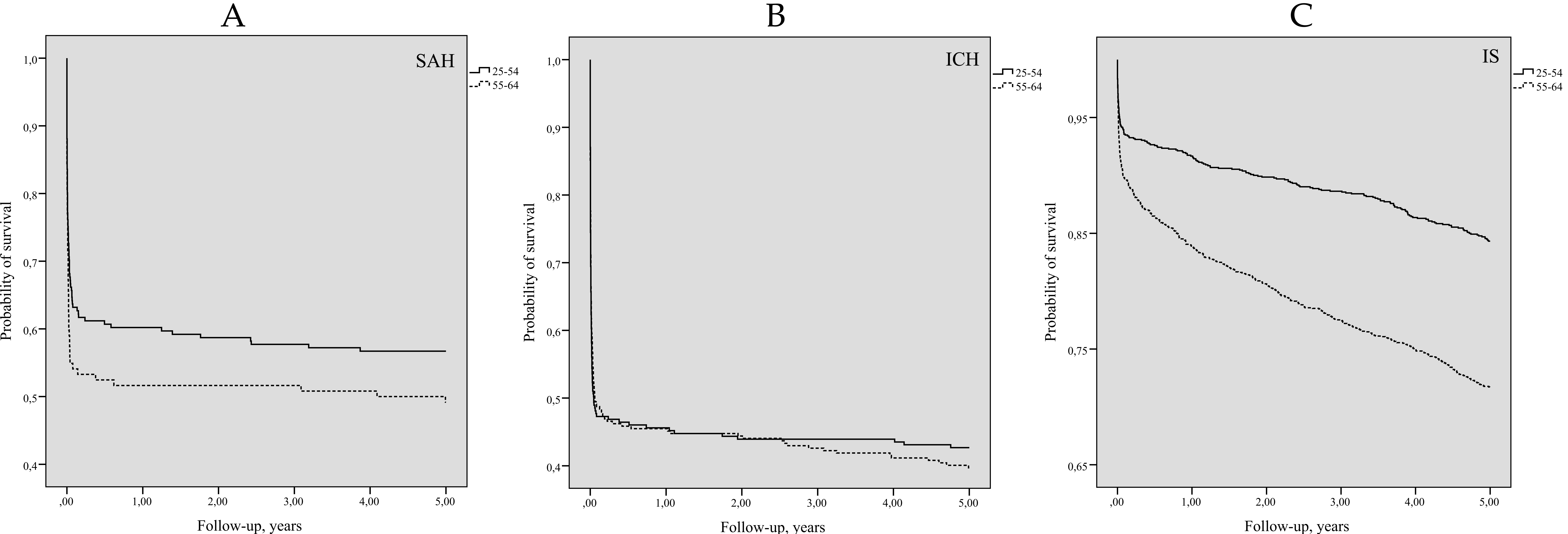

Supplement: S5 Fig — Kaplan-Meier 5-year survival curves for persons with subarachnoidal hemorraghe (A), ischemic cerebral hemorraghe (B) and ischemic stroke (C) by age. (A) Log-rank = 2.015, p = 0.156, (B) Log-rank = 0.111, p = 0.739, (C) Log-rank = 69.384, p = 0.0001 (TIF) [file pone.0219392.s005.tif]

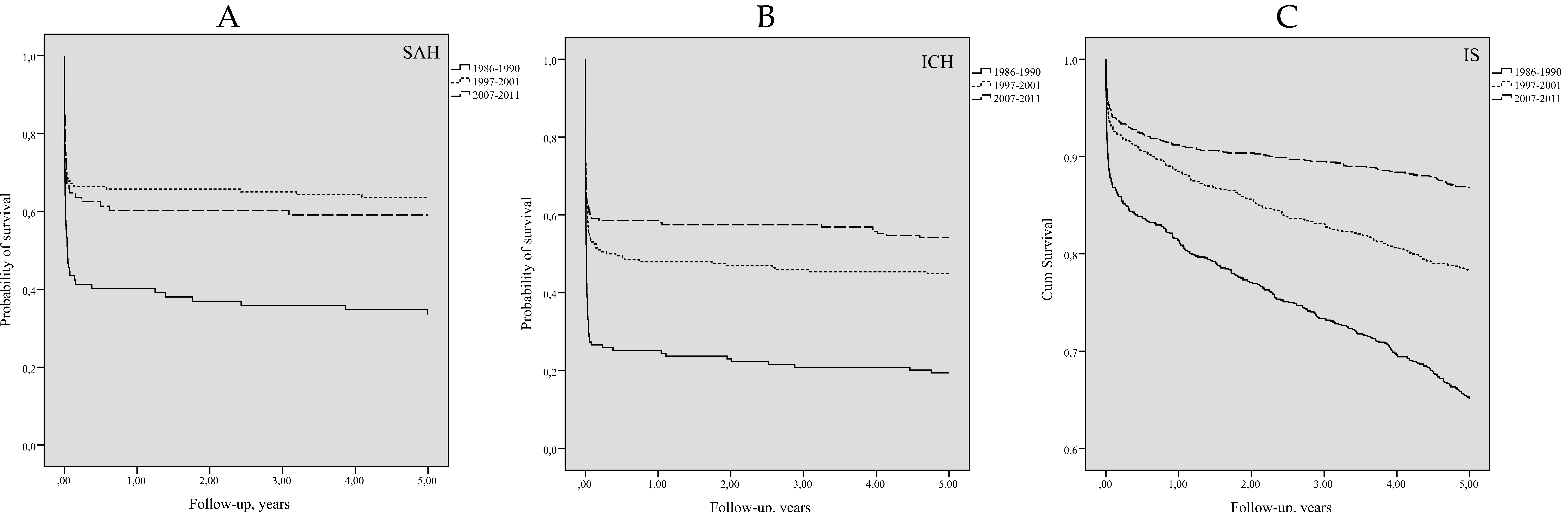

Supplement: S6 Fig — Kaplan-Meier 5-year survival curves for persons with subarachnoidal hemorraghe (A), ischemic cerebral hemorraghe (B) and ischemic stroke (C) by time cohort. (A) Log-rank = 19.133, p = 0.0001; comparing 1986–1990 with 1997–2001, (A) Log-rank = 11.828, p = 0.001; comparing 1986–1990 with 2007–2011 (A) Log-rank = 0.260, p = 0.610; comparing 1997–2001 with 2007–2011 (B) Log-rank = 24.050, p = 0.0001; comparing 1986–1990 with 1997–2001, (B) Log-rank = 36.808, p = 0.0001; comparing 1986–1990 with 2007–2011 (B) Log-rank = 2.677, p = 0.102; comparing 1997–2001 with 2007–2011 (C) Log-rank = 46.546, p = 0.0001; comparing 1986–1990 with 1997–2001, (C) Log-rank = 133.720, p = 0.0001; comparing 1986–1990 with 2007–2011 (C) Log-rank = 26.098, p = 0.0001; comparing 1997–2001 with 2007–2011 (TIF) [file pone.0219392.s006.tif]
